# Rapid functional and evolutionary changes follow gene duplication in yeast

**DOI:** 10.1098/rspb.2017.1393

**Published:** 2017-08-23

**Authors:** Samina Naseeb, Ryan M. Ames, Daniela Delneri, Simon C. Lovell

**Affiliations:** School of Biological Sciences, Faculty of Biology, Medicine and Health, University of Manchester, Oxford Road, Manchester M13 9PT, UK

**Keywords:** gene duplication, evolution, functional innovation, gene expression

## Abstract

Duplication of genes or genomes provides the raw material for evolutionary innovation. After duplication a gene may be lost, recombine with another gene, have its function modified or be retained in an unaltered state. The fate of duplication is usually studied by comparing extant genomes and reconstructing the most likely ancestral states. Valuable as this approach is, it may miss the most rapid evolutionary events. Here, we engineered strains of *Saccharomyces cerevisiae* carrying tandem and non-tandem duplications of the singleton gene *IFA38* to monitor (i) the fate of the duplicates in different conditions, including time scale and asymmetry of gene loss, and (ii) the changes in fitness and transcriptome of the strains immediately after duplication and after experimental evolution. We found that the duplication brings widespread transcriptional changes, but a fitness advantage is only present in fermentable media. In respiratory conditions, the yeast strains consistently lose the non-tandem *IFA38* gene copy in a surprisingly short time, within only a few generations. This gene loss appears to be asymmetric and dependent on genome location, since the original *IFA38* copy and the tandem duplicate are retained. Overall, this work shows for the first time that gene loss can be extremely rapid and context dependent.

## 1. Introduction

Gene duplication can significantly speed up evolution by providing new redundant genetic material that has no constraints and can freely evolve new functions [1]. Duplicates can also confer an immediate fitness benefit when an increased gene dosage is advantageous [2,3]. Ancestral functions may be partitioned between duplicates (subfunctionalization) [4], or duplicate copies may acquire new functions (neofunctionalization) [5]. A duplicate can also recombine with another gene to form a chimeric gene leading to innovation of gene function [6,7]; indeed chimeric genes are found in natural yeast hybrids [8]. However, since genetic redundancy is not a selective trait *per se,* the fate of the majority of duplicate gene copies is to be lost from the genome.

After a whole genome duplication (WGD) event in yeast approximately 88% of duplicated genes were lost over a period of 100 million years [9], and yeast species display a large turnover of duplicate genes [10]. There may be selection pressure to remove a duplicate if it results in an imbalance of protein subunits in a protein complex [11], and duplicate retention may be influenced by selection from the environment for specific functions [12].

Numerous mechanisms have been proposed to explain the retention and loss of duplicate genes. Neutral mechanisms affect both retention and loss of duplicates through subfunctionalization and pseudogenization respectively [4,13]. The environment [12,14], scale of duplication [15] and location of the duplication event [16] may also influence duplicate retention. However, the comparative genomics approaches that are used to study gene duplication are inevitably retrospective. In particular, rapid changes are difficult to detect and identification would require high-density sampling of strains at a time relevant to the duplication.

We investigated the most rapid mechanisms that govern the retention or loss of duplicate genes by introducing an artificial duplicate into the genome of *Saccharomyces cerevisiae*. The study of an artificial duplicate in yeast allows us to test whether there is an immediate fitness benefit after duplication, and the molecular mechanism by which a benefit may arise. Allowing the duplicate strains to evolve in different environments will allow us to test whether environmental selection plays a role in duplicate retention. Furthermore, we can test for expression and fitness differences between ancestral and evolved strains. Finally, by introducing duplicates in tandem and non-tandem positions we can test the effects of location on duplicate retention and organismal fitness. We chose to duplicate a singleton gene (i.e. without confounding effect of paralogues) that is highly conserved among eukaryotes and has a large number of genetic and physical interactions (i.e. so that duplication is more likely to trigger measurable fitness changes). *IFA38*, which encodes for an elongase enzyme required for very long-chain fatty acid synthesis, has 104 interactions (ranked top 10 orthologous interacting protein in yeast) and is highly conserved [17]. *IFA38* is important for maintenance of membrane fluidity [18] and for resistance to ethanol and other stressors [19]. Hence, fitness output of engineered duplicate strains can be easily scored on media containing ethanol. Similarly, we can also test the fitness under non-fermentable conditions by using glycerol as a carbon source, which is exclusively respired by yeast and so provides an ethanol-free environment.

We find that introduction of an extra copy of *IFA38* triggers a global transcriptional response and can confer a fitness benefit, although the magnitude of this benefit depends on both the genomic location of the duplicated gene and the environment. We also show that a gene duplicate can be lost from the genome very rapidly under respiratory conditions, and the loss is asymmetric (i.e. deletion of the newly duplicated copy). Overall, evolutionary changes in response to duplication of *IFA38* gene can be extremely fast and modulated by the environment and genomic context.

## 2. Material and methods

### (a) Strains, media and culture conditions

The parental strain used in these experiments is the standard laboratory strain of *S. cerevisiae* BY4743 (*MAT*a/α *his3*Δ*1/his3*Δ*1 leu2*Δ*0/leu2*Δ*0 LYS2/lys2*Δ*0 met15*Δ*0/MET15 ura3*Δ*0/ura3*Δ*0*). All the strains were maintained on YPD medium (1% (w/v) yeast extract, 1% (w/v) peptone and 2% (w/v) glucose) containing required antibiotics: 300 µg/ml geneticin (GibcoBRL) for selection of the *kanMX* markers. YP + glycerol medium was prepared by supplementing YP medium with 2% (w/v) glycerol, and ethanol-containing medium was prepared by supplementing YPD with 5% (w/v) or 7% (w/v) ethanol as per requirement.

### (b) Genetic engineering of strains possessing duplicated genes

To construct the strains possessing duplicate genes, a resistance marker cassette (*loxP-kanMX-loxP*) was inserted at the downstream region of the gene of interest (in this study *IFA38*) in *S. cerevisiae* (BY4743) using PCR-mediated gene replacement mechanism [20,21] and the standard lithium acetate transformation method [22]. Correct transformants were confirmed by analytical PCR. All the primers used in this work are provided in electronic supplementary material, tables S1 and S2.

To distinguish the original *IFA38* gene from its duplicate copy, up to five differences in the sequence were introduced in the duplicates, without altering the protein sequence and the codon adaptation index, as measured by CodonW (electronic supplementary material, table S3). These differences also do not disrupt any known transcription factor binding sites as identified in the Yeastract database [23].

### (c) Fitness assays

The competitive fitness of ancestral and evolved cultures versus the GFP tagged reference strains was measured by a FACS based competition assay as described previously [24–26]. Growth was also tested in monocultures using FLUOstar optima microplate reader in YPD, YPD + 7% ethanol and YP + 2% glycerol media as previously described [27,28].

### (d) Experimental evolution

Five independent biological replicates of the mutants (tandem and non-tandem duplicates) and WT strain were allowed to evolve for 500 generations under three different conditions (YPD, YPD + 5% ethanol and YP + 2% glycerol) with shaking at 30°C. Overnight grown strains were washed with sterile water and the cell count was taken using cellometer auto M10 (Peqlab). Approximately 1 × 10^6^ cells were transferred in the 96 well plate containing 200 µl of the respective medium. The cultures evolved in YPD and YPD + 5% ethanol environments were transferred into fresh media after every 24 hours, whereas the YP + 2% glycerol-evolved ones were transferred after 48 h. Overall, 500 generations were achieved in five, seven and nine months in YPD, YPD + 7% ethanol, and YP + 2% glycerol medium, respectively.

### (e) DNA extraction and whole genome sequencing

Total genomic DNA was extracted from overnight grown culture of yeast strains using the standard phenol/chloroform method [29]. Paired end whole-genome sequencing was performed using the Illumina HiSeq platform. Quality control was applied to sequence reads using FastQC (Babraham Bioinformatics), reads were aligned using Bowtie2 [30] and post-processed using samtools [31]. Single nucleotide polymorphisms (SNPs) were identified using the Genome Analysis ToolKit (GATK) [32] and genes containing SNPs were tested for enrichment of GO terms [33]. Full details and all parameter settings can be found in electronic supplementary material, File S1.

### (f) RNA extraction, reverse transcription and real-time quantitative PCR

Total RNA was extracted in YPD, YPD + 7% ethanol and YP + 2% glycerol media by either using Qiagen RNeasy Mini kit for real time PCR experiments (catalogue no. 74104) or by using Trizol reagent for RNA sequencing (Invitrogen, catalogue no. 155-96-018). cDNA from total RNA was synthesized using Qiagen reverse transcription kit (catalogue no. 205311). The expression level of *IFA38* was determined using quantitative real-time PCR as described previously [34]. The primers used for the real-time PCR are in electronic supplementary material, table S4.

### (g) RNA-Seq and copy number variant analysis

1–4 µg of total RNA was processed for RNAseq using the illumina HiSeq 2500. RNA-Seq reads were aligned with Bowtie2 [30] and resulting alignment files were processed with samtools [31]. HT-Seq [35] was used for counting reads mapping to known genes and edgeR was used to identify differentially expressed (DE) genes, which were tested for enrichment of GO terms [33]. Finally, CNV-Seq [36] was used to identify copy number variants. Full details and all parameter settings can be found in electronic supplementary material, File S1. Raw sequence reads are available in the Sequence Read Archive under accession SRP074528.

## 3. Results

### (a) Construction of duplicated strains

Strains possessing a duplicate copy of the *IFA38* gene were successfully constructed using the *cre-loxP* system [28,34,37–39]. The transcript boundaries (3′ and 5′ UTRs) of *IFA38* were obtained from a previously published study [40] and the gene was amplified along with its UTRs (electronic supplementary material, figure S1*a*), such that all regulatory sequences were also duplicated. New gene copies tagged with a resistance marker cassette (KanMX) were inserted at tandem and at non-tandem positions in *S. cerevisiae* BY4743 background (electronic supplementary material, figure S1*b–f*). In the text, the tandem and non-tandem strains will be referred to as ‘IFA38-t’ and ‘IFA38-nt’ respectively. The tandem duplication is located approximately 150 bp downstream of the 3′UTR of *IFA38* gene (insertion coordinate 559878). The non-tandem duplication is located nearer to the centromere, approximately 310 kb away from *IFA38* (insertion coordinate 248803). Expression at centromeres is known to be different from the remainder of the genome and is epigenetically regulated [41]. Various transcription factors such as Cbf1 and Ste12 and silencing factors are known to contribute to the transcriptional regulation at centromere [42]. This can potentially affect the level of expression of the newly inserted gene duplicate and eventually its retention. Neither duplication disrupts known transcription factor binding sites as identified in the Yeastract database [23], nor is either duplication near a transposable element, the closest being 11 000 base pairs away.

### (b) Gene duplication can confer a fitness advantage immediately after duplication

To determine whether the duplication of *IFA38* conferred a fitness advantage immediately after duplication we performed competition assays in three different media: standard rich medium YPD; YPD + 7% ethanol; and YP + 2% glycerol. In YPD, cells can both ferment and respire, and although ethanol is not present at the beginning of the experiment, it can be produced by the fermentative action of the yeast strains. YPD containing 7% ethanol has been used as selective medium to test the fitness of our duplicated strains, since the deletion of *IFA38* causes a significant decrease in growth in rich medium containing ethanol (electronic supplementary material, figure S2). Lastly, we used YP + 2% glycerol medium, which has glycerol as the sole carbon source, restricting the yeast to respiration. Here, we expect that there will be no selection pressure to retain the additional copy of *IFA38*, since ethanol is not present.

**Figure 1.**
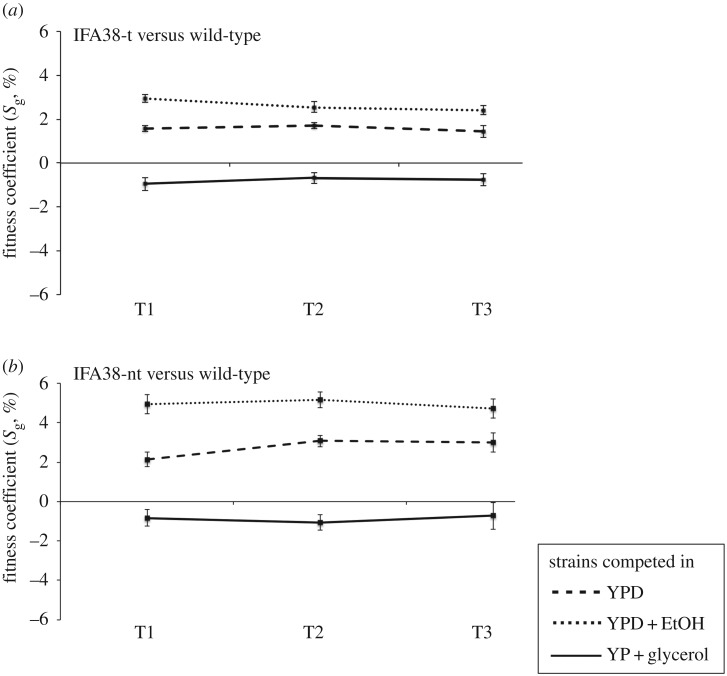
Competitive fitness of ancestral strains in different media conditions. Fitness coefficients of (*a*) tandem ‘IFA38-t’ and (*b*) non-tandem ‘IFA38-nt’ duplicates relative to the wild-type strain competed in YPD (broken line), YPD + 7% ethanol (dotted line) and YP + 2% glycerol (solid line) medium. Both strains show an increase in fitness in YPD and YPD + 7% ethanol-containing medium, whereas reduced growth was observed in the YP + 2% glycerol environment. T1, T2 and T3 represent three different points of cell count after every 10 generations. The error bars represent the average of three technical replicas of five independent biological replicas. Error bars are at 95% CIs.

Immediately after the duplication, we find that both the tandem and non-tandem duplications confer a significant fitness benefit over the wild-type strain in YPD medium (*p* < 0.01, *t*-test; figure 1). When the strains are competed in YPD + 7% ethanol-containing medium we see a much higher increase in fitness of both tandem and non-tandem duplicate strains (*p* < 0.01, *t*-test; figure 1); on the other hand, when growing the cells on in YP + 2% glycerol there is a small but significant decrease in fitness for the duplicate strains (*p* < 0.05, *t*-test; figure 1). Moreover, the fitness of intermediate control strain lacking duplication but containing *loxP-KanMX-loxP* was also tested and no fitness differences were observed compared with the wild-type strain (data not shown). These results suggest that following the duplication event the presence of an extra copy of *IFA38* confers a growth advantage over the wild-type strain in rich YPD media with or without ethanol.

### (c) Gene duplication results in increased gene expression in certain environments

When the duplicated strains are grown on YPD or YP + 2% glycerol media, the expression levels for *IFA38* are similar to the wild-type (electronic supplementary material, figure S3*a*,*c*). Interestingly, despite the *IFA38* being expressed at a similar level in YPD and YP + 2% glycerol media, the duplication gives only a fitness advantage in YPD (figure 1). However, in YPD + 7% ethanol medium we see a significant increase in expression of *IFA38* in both types of duplicate strains (electronic supplementary material, figure S3*b*), with the non-tandem duplicate strain showing a larger increase in expression compared to the wild-type than the tandem duplicate strain. *IFA38* is therefore upregulated in media containing ethanol and its overall expression in the duplicated strains is increased compared with the wild-type.

### (d) Evolution under different environmental conditions affects fitness

To examine the long-term fitness effects and other evolutionary changes due to the presence of a duplicate gene, our duplicate strains were serially sub-cultured for 500 generations in YPD, YPD + 5% ethanol and YP + 2% glycerol. Competitive fitness of evolved versus ancestral population was measured in YPD, YPD + 7% ethanol and YP + 2% glycerol.

When fitness was measured in YPD, all populations of wild-type and duplicate strains evolved in YPD and YPD + 5% ethanol showed a final increase in growth compared with the ancestral populations (electronic supplementary material, figure S4*a–c*, broken and dotted lines), whereas no change of competitive fitness was observed for the yeast population evolved in YP + 2% glycerol (electronic supplementary material, figure S4*a–c*, solid lines).

Competitive fitness tested in YPD + 7% ethanol of all populations evolved in YPD + 5% ethanol was increased compared with the respective ancestral populations (*p* < 0.01; electronic supplementary material, figure S5*a–c*, dotted lines), while the opposite is true for all the populations evolved in YP + 2% glycerol (electronic supplementary material, figure S5*a–c*, solid lines). For the yeast cultures evolved in YPD, a significant decrease in growth was seen only for the strains carrying the duplications when compared with their respective ancestral strains (Student's *t*-test, *p* < 0.05; electronic supplementary material, figure S5*b*,*c*, broken lines).

When competitive fitness of the evolved populations is measured in YP + 2% glycerol medium, none of the populations of duplicate strains (electronic supplementary material, figure S6*b*,*c*) show fitness differences compared with the ancestral populations, except for the tandem duplication evolved in YP + 2% glycerol, which shows a decrease in fitness (electronic supplementary material, figure S6*b*, solid line). Overall, these data show differences in competitive fitness of the evolved populations based on the medium.

### (e) Expression of *IFA38* in the evolved populations

**Figure 2.**
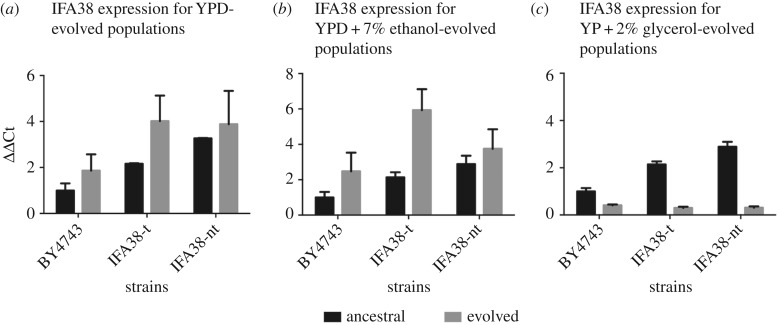
The expression of *IFA38* of the wild-type and duplicate strains before and after evolution in YPD + 7% ethanol media. The strains were evolved in (*a*) YPD, (*b*) YPD + 7% ethanol and (*c*) YP + 2% glycerol. The bar charts show the *IFA38* expression at the start of the evolution experiment (black bars) and after evolution at 500th generation (grey bars). Error bars are from three technical replicas for each of the five independent biological samples. Relative normalized fold expression was calculated by using ΔΔCt method and *ACT1* was used as a reference gene.

We assessed the expression of *IFA38* in YPD + 7% ethanol for all the evolved populations. When yeast strains are allowed to evolve in YPD, all strains including the wild-type show increased expression of *IFA38* after 500 generations (figure 2*a*). We see the same trend for the strains evolved in YPD + 5% ethanol (figure 2*b*), and in the case of the tandem duplicate the expression after experimental evolution is three times higher than the initial one. The strains carrying the duplication were also evolved in YP + 2% glycerol medium, where the presence of an extra copy of *IFA38* had lower fitness. For these strains, when the level of transcription of *IFA38* was measured in YPD + 7% ethanol, we see a drastic reduction in the expression in all the evolved strains (figure 2*c*). This suggests that long-term growth in an environment containing glycerol represses the expression of *IFA38*, while long-term growth in YPD and in ethanol-containing media increases it.

### (f) Global changes in gene expression after duplication and evolution

To determine whether fitness differences associated with the duplication arise only from altered expression of *IFA38* or instead are due to global transcriptomic changes brought about by the introduction of the *IFA38* duplicate, we carried out RNA-Seq experiments for ancestral and evolved strains. We found that transcriptional changes immediately after duplication and after long-term evolution are not only confined to *IFA38* but are widespread throughout the genome.

Immediately after duplication, by comparing the ancestral wild-type strain with the duplicate strains, we can identify a total of 2597 (50.8%) and 2239 (43.8%) genes significantly differentially expressed in the tandem and non-tandem duplication strains, respectively. The duplication of a gene with a high number of genetic and physical interactions, such as *IFA38*, can therefore greatly alter the transcriptome immediately after the introduction of the gene.

**Figure 3.**
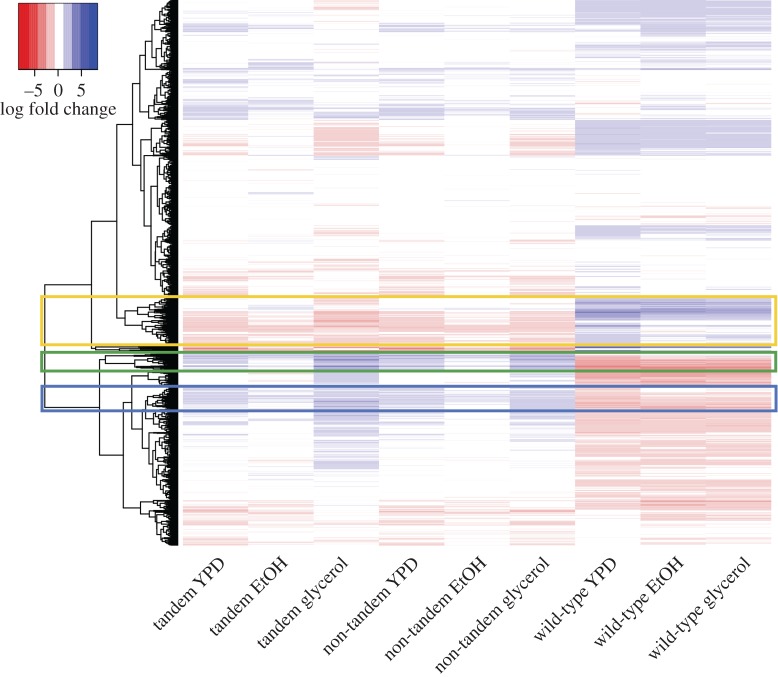
Differential expression of genes after experimental evolution. Each row represents a gene, and the ordering of the dendrogram and rows is inferred from hierarchical clustering of the expression data. Increased and decreased expression is indicated by log-fold change and shown in blue and red, respectively. The green and blue highlighted sections identify groups of genes up- and down-regulated in the duplicate and wild-type strains, respectively. The gold highlighted section shows a group of genes down- and up-regulated in the duplicate and wild-type strains, respectively.

We then identified significantly DE genes after 500 generations and observed that expression changes occur after evolution in specific environments (figure 3). Interestingly, we can see almost opposite changes in expression between the duplicate strains and the wild-type strains after evolution (figure 3). For example, compared with the ancestral strains, we observe a significant reduction of expression in enzymes linked to the ethanol pathway, such as *ADH2* and *ALD2*, in all duplicate strains, but not in the wild-type, where *ADH2* expression increases. A reduced expression of both *GUT1* and *GUT2*, enzymes in the glycerol degradation pathway, is also detected for all the strains carrying duplications, while an opposite trend is seen for the WT strain.

Identifying genes that have altered expression after 500 generations allows us to detect the types of functions important for evolution in a particular environment. Specifically, we identify the types of genes consistently upregulated in duplicate strains but downregulated in wild-type strains after experimental evolution (green highlighted region in figure 3). This group of genes is enriched for gene ontology (GO) terms associated with sugar transport and metabolism. Another cluster of genes upregulated in the duplicate strains but downregulated in the wild-types is enriched for GO terms associated with amino acid biosynthesis and other translation associated terms (blue highlighted region in figure 3). Conversely, genes that are consistently downregulated in duplicate strains and upregulated in wild-type strains (gold highlighted region in figure 3) are enriched for GO terms related to fatty acid and lipid catabolism. A complete list of enriched GO terms in these regions can be found in electronic supplementary material, table S5.

We can also identify some common functions for upregulated genes such as those involved in carbohydrate transport which are over-expressed in all strains evolved in an environment containing ethanol (electronic supplementary material, table S6). The high-affinity glucose transporter, *HXT6*, has been shown previously to be upregulated in cells growing on non-fermentable carbon sources such as ethanol [43], and the production of storage carbohydrates has been identified as part of the yeast environmental stress response [44,45]. We conclude that the duplication of *IFA38*, which possesses a high number of interactions, has the potential to drastically alter the evolutionary trajectory of a strain.

### (g) Single nucleotide polymorphisms arising during experimental evolution

We identified SNPs causing missense mutations in genes during experimental evolution (electronic supplementary material, table S7). We can identify SNPs with possible relevance to environmental adaptation; in the wild-type strain evolved in YPD we can detect an SNP in *ELO1*, another gene involved in fatty acid chain elongation. We can also identify genes with SNPs that occur in multiple strains; *HXT* genes involved in hexose transport show SNPs in multiple strains, as do genes associated with ATPase activity (*ENA1* and *ENA2*), cell wall integrity (*ASP3* and *MKK1*) and elongation factors associated with translation (*EFT1/2* and *TEF1*). However, none of the genes with SNPs identified in our GATK analysis have any known physical interactions with *IFA38*.

Several genes in the duplicate strains accumulate mutations independently in all three environments, although the type and position of these SNPs vary across strains (electronic supplementary material, figure S7). Both tandem and non-tandem duplicate strains had a higher number of shared genes among the different environments when compared with the wild-type strain. GO was used to characterize the functions of genes that accumulate SNPs in multiple environments. We detected enrichment for sugar transporters in our tandem duplicate, suggesting a common evolutionary trajectory for these strains.

We also identify SNPs in similar types of genes across the strains. We find GO terms related to transmembrane transport, hexose transport and translation elongation enriched for genes with SNPs in multiple strains (electronic supplementary material, table S8). This evolutionary trend for transmembrane transporters was also detected in our transcriptomics work (i.e. significant change in expression pattern).

### (h) Detection of duplicate loss during experimental evolution

To determine whether there had been any subsequent changes in copy number of *IFA38* after duplication and evolution we used CNV-Seq to compare the read-depth of *IFA38* in the sequencing data between ancestral and evolved strains. For strains evolved in YPD and ethanol there appear to be no copy number changes of *IFA38* after 500 generations. However, there was a reduction in copy number of *IFA38* in the non-tandem strain evolved in glycerol, highlighted by a relative reduction in read-depth in the region of *IFA38* compared with the ancestral strain (CNV-Seq *p* < 0.01; electronic supplementary material, figure S8).

**Figure 4.**
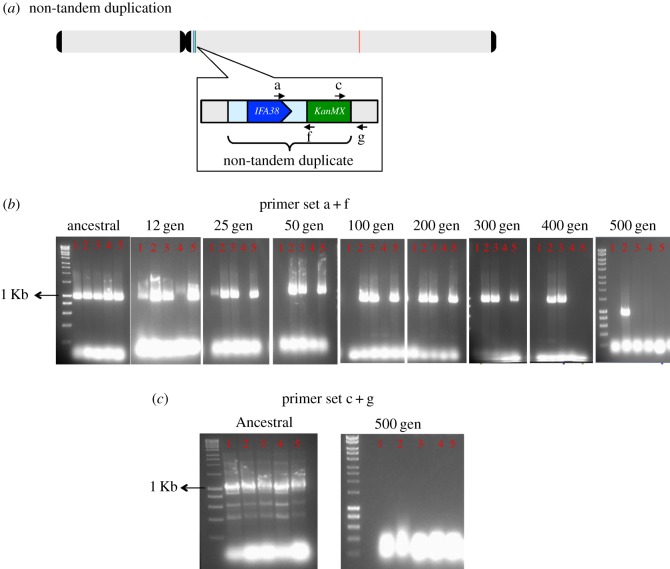
Detection of loss of the engineered *IFA38* duplicate via analytical PCR. The presence and loss of *IFA38* duplicate was confirmed by PCR. (*a*) A diagrammatic view of the original gene (red) and engineered copy (blue) of *IFA38* on the chromosome and the position of the primers (a,f,c,g; black arrows) used for PCR. (*b*) 1.5% (w/v) agarose gel representing the duplicate colonies of ancestral and glycerol-evolved strains confirmed by primers a + f giving a product of expected band size of 1008 bp. (*c*) 1.5% (w/v) agarose gel representing the duplicate colonies of ancestral and glycerol-evolved strains confirmed by primers c + g giving a product of expected band size 1032 bp.

We experimentally validated these predicted losses and looked for (i) further evidence of duplicate loss in all biological replicates of glycerol-evolved strains, (ii) the asymmetry of gene loss (i.e. deletion of the duplicate or original copy) and (iii) the time scale of the loss. Analytical PCR using the primers specific to the original gene showed that the *IFA38* was retained in all the biological replicates of non-tandem duplicates after evolving them in glycerol medium for 500 generations (electronic supplementary material, figure S9*b*,*c*), suggesting that it is the duplicate that is lost. To understand if the engineered copy of *IFA38* was retained or lost during the period of evolution, analytical PCR was performed using the primers specific to the engineered gene and the marker cassette (figure 4*a*). We found that the engineered copy was lost from four out of five biological replicates after 500 generations (figure 4*b*,*c*). In addition to this, the event of gene loss was traced back to earlier generations, namely after 400, 300, 200, 100, 50, 25 and 12 cell divisions. It was found that the four biological replicates lost the duplicate genes at different stages: biological 4, 1, 5 and 3 lost the *IFA38* duplicate between 12–25, 25–50 and 400–500 generations, respectively (figure 4*b*). The single replicate (biological 2) from which the engineered copy was not lost after 500 generations had a partial deletion of the duplication cassette (figure 4*c*). Taken together, these data show for the first time that loss of duplicates is extremely rapid. Interestingly, former studies have shown gain of new duplicate genes in *E. coli* and yeast strains after evolving them in a glucose-limited environment [6,46].

## 4. Discussion

In this work, we addressed the very earliest events of post-duplication using an experimental approach to determine the immediate changes in expression and cellular fitness. Such an approach limits the number of genes and genomic positions that can be studied, but has the advantage of allowing insights into both the quantitative molecular changes and the very first fitness effects that arise from duplication, and so complements traditional computational approaches. Since biases at these very early stages have the potential to influence patterns of retention and innovation observed later, identifying and understanding them is essential in producing a complete picture of the fate of gene duplicates.

Gene duplication obviously alters gene dosage, which may in turn alter the amount of protein present. In yeast, 80% of genes have a strong correlation between copy number and expression [47]. However, in the longer term expression can change [48], and neutral changes in expression can give rise to subfunctionalisation [49]. Our results show that, upon duplication, widespread expression changes occur and are not limited to the duplicated gene.

Immediately after duplication of *IFA38* there is an increase in expression, and this increase is dependent on both growth conditions and the genomic context of the duplication (electronic supplementary material, figure S3). It is possible that the genomic location where the non-tandem duplicate was inserted is more accessible to transcriptional changes. Indeed, different genomic regions have markedly different levels of expression [50], with genes located near the telomeres being repressed [51].

We find that changes in expression that arise from duplication are rapidly accommodated, although this also depends on growth conditions and genomic context. Expression changes are additionally observed in a large number of other genes, immediately after duplication and over the course of experimental evolution (figure 3). Here, we can see that approximately 50% of genes show significant DE in both tandem and non-tandem duplication strains, suggesting that even single gene duplications may perturb the system as much as environmental changes. Previous studies in *Drosophila* have shown that new genes can evolve rapidly and result in extensive gene network changes on short evolutionary time scales [52–55]. Moreover, many duplications can cause downstream global changes, but most often with negative fitness effects, therefore subject to purifying selection they are eliminated quickly [56]. We observed a reversed pattern of expression between the strains carrying the *IFA38* duplication and the WT for key genes in the fatty acid and lipid catabolism, sugar transport, ethanol and glycerol utilization, and amino acid biosynthesis pathways.

We also observe common patterns of SNPs. We find that the same and functionally similar genes accumulate SNPs across duplicate strains and environments. These include SNPs in genes related to transmembrane transport, sugar transport and translation elongation that may represent common adaptations to these environments that can arise regardless of the duplication event. Indeed, the wild-type strains accumulate SNPs in genes associated with ATPase activity and translation elongation. Not all the SNPs identified in this study will play a role in environmental adaptation; in fact only a minority of mutations persist in the population and may act as drivers of adaptation [57].

When the duplicate strains are competed in media containing glycerol we see a decrease in fitness, whereas in ethanol-containing media we see an immediate increase in fitness following the duplication (figure 1). This suggests a potential gene dosage benefit of *IFA38*, perhaps to respond to any ethanol produced from glucose fermentation in YPD media [58,59].

In our evolved populations, the growth of glycerol-evolved strains remained unaltered in YPD medium (electronic supplementary material, figure S4, solid line). By contrast, the glycerol-evolved duplicates were less fit relative to the ancestral duplicates in the ethanol-containing medium and the YPD (electronic supplementary material, figure S5*b*,*c*) [60]. The wild-type strain after evolution in ethanol medium showed increased fitness, demonstrating positive selection in that particular environment.

We find that gene loss can happen much more rapidly than previously appreciated, with the deletion of four out of five non-tandem artificial duplicates within 500 generations, with the first loss detected after 25 generations. However, all the strains carrying tandem duplication retained both the inserted and the original copy of the gene. A previous theoretical study has shown that rate of gene loss is independent of gene linkage and occurs at same order of magnitude in both tandem and non-tandem duplications [61]. In our case the non-tandem copy is lost more easily in glycerol, and we detect a genomic location effect. Such an effect could be due to the fact that the insertion is in the proximity of the centromere, since it is known that expression at centromeres is different from the remainder of the genome [41]. It is possible that increased expression of *IFA38* from the addition of a duplicate copy could have led to the duplicate being lost, as increased gene expression may have a negative effect on fitness [11,62,63]. However, in an environment containing glycerol there is no evident increase in expression of *IFA38* after duplication (electronic supplementary material, figure S3*c*). The duplicates and wild-type strains evolved in YPD and YPD + 5% ethanol showed an increased expression of *IFA38* in the YPD + 7% ethanol, whereas the glycerol-evolved strains when grown in ethanol showed a reduction of *IFA38* expression in all evolved strains compared with the ancestral. This demonstrates that non-functionalization may occur at the expression level before changes in the coding sequence create faulty proteins.

Our results reconcile the apparent difference between the immediate [47] and longer-term [48,49] effects of duplication as we see both effects in our experiment. Since the effects of the duplication are contingent both on genomic position and growth environment, our results also offer an explanation of why evolutionary trends of retention ascribed to dosage and stoichiometric balance are significant, but not universal [15,49,64]. After the yeast WGD, gene loss is known to be rapid, but the true initial rate is difficult to measure with any accuracy [65]. The extremely rapid loss of the duplicated gene we observe here happens so quickly that neither the duplication nor the loss can be observed by previous computational studies [13].

Importantly, in all cases the original copy of *IFA38* was maintained, and the duplicated gene is the one lost. This rapid and asymmetric loss suggests that there is selection for the duplicate to be removed, although any fitness difference between the wild-type and the strain carrying the duplicate must be too small to be measured in the competition experiment in the glycerol conditions. A previous study on duplicate loss after WGD event found that orthologues are retained more frequently than paralogues, suggesting that at least some duplicate pairs are not functionally equivalent to each other [65]. Genomic position can affect biased duplicate loss [16], which may be due to DE in different regions of the genome caused by chromatin binding, or other constraints on recombination that are genome-context-specific. Given the importance of environmental conditions for determining duplicate loss or retention, the set of genes lost or retained in one growth condition may limit an organism in its ability to colonize other environments. Condition-specific gene loss may therefore be an early contributor to speciation.

## Supplementary Material

Supplementary file 1

## Supplementary Material

Supplementary Figures and tables Legends

## Supplementary Material

Figure S1

## Supplementary Material

Figure S2

## Supplementary Material

Figure S3

## Supplementary Material

Figure S4

## Supplementary Material

Figure S5

## Supplementary Material

Figure S6

## Supplementary Material

Figure S7

## Supplementary Material

Figure S8

## Supplementary Material

Figure S9

## Supplementary Material

Table S1

## Supplementary Material

Table S2

## Supplementary Material

Table S3

## Supplementary Material

Table S4

## Supplementary Material

Table S5

## Supplementary Material

Table S6

## Supplementary Material

Table S7

## Supplementary Material

Table S8

## Data accessibility

Raw sequence reads for this study are available in the Sequence Read Archive under accession SRP074528.

## References

[1] Ohno S 1970 Evolution by gene duplication. New York, NY: Springer.

[2] Otto SP, Whitton J 2000 Polyploid incidence and evolution. Annu. Rev. Genet. 34, 401–437. (10.1146/annurev.genet.34.1.401) 11092833

[3] Conant GC, Wolfe KH 2007 Increased glycolytic flux as an outcome of whole-genome duplication in yeast. Mol. Syst. Biol. 3, 129–141. (10.1038/msb4100170) 17667951 PMC1943425

[4] Force A, Lynch M, Pickett FB, Amores A, Yan Y, Postlethwait J 1999 Preservation of duplicate genes by complementary, degenerative mutations. Genetics 151, 1531–1545. 10101175 10.1093/genetics/151.4.1531 PMC1460548

[5] Assis R, Bachtrog D 2013 Neofunctionalization of young duplicate genes in *Drosophila*. Proc. Natl Acad. Sci. USA 110, 17 409–17 414. (10.1073/pnas.1313759110) PMC3808614 24101476

[6] Brown CJ, Todd KM, Rosenzweig RF 1998 Multiple duplications of yeast hexose transport genes in response to selection in a glucose-limited environment. Mol. Biol. Evol. 15, 931–942. (10.1093/oxfordjournals.molbev.a026009) 9718721

[7] Long M, Betran E, Thornton K, Wang W 2003 The origin of new genes: glimpses from the young and old. Nat. Rev. Genet. 4, 865–875. (10.1038/nrg1204) 14634634

[8] Hewitt SK, Donaldson IJ, Lovell SC, Delneri D 2014 Sequencing and characterisation of rearrangements in three *S. pastorianus* strains reveals the presence of chimeric genes and gives evidence of breakpoint reuse. PLoS ONE 9, e92203 (10.1371/journal.pone.0092203) 24643015 PMC3958482

[9] Kellis M, Birren BW, Lander ES 2004 Proof and evolutionary analysis of ancient genome duplication in the yeast *Saccharomyces cerevisiae*. Nature 428, 617–624. (10.1038/nature02424) 15004568

[10] Ames RM, Money D, Lovell SC 2014 Inferring gene family histories in yeast identifies lineage specific expansions. PLoS ONE 9, e99480 (10.1371/journal.pone.0099480) 24921666 PMC4055711

[11] Papp B, Pal C, Hurst LD 2003 Dosage sensitivity and the evolution of gene families in yeast. Nature 424, 194–197. (10.1038/nature01771) 12853957

[12] Ames RM, Rash BM, Hentges KE, Robertson DL, Delneri D, Lovell SC 2010 Gene duplication and environmental adaptation within yeast populations. Genome Biol. Evol. 2, 591–601. (10.1093/gbe/evq043) 20660110 PMC2997561

[13] Lynch M, Conery JS 2000 The evolutionary fate and consequences of duplicate genes. Science 290, 1151–1155. (10.1126/science.290.5494.1151) 11073452

[14] Harrison R, Papp B, Pal C, Oliver SG, Delneri D 2007 Plasticity of genetic interactions in metabolic networks of yeast. Proc. Natl Acad. Sci. USA 104, 2307–2312. (10.1073/pnas.0607153104) 17284612 PMC1892960

[15] Hakes L, Pinney J, Lovell S, Oliver S, Robertson D 2007 All duplicates are not equal: the difference between small-scale and genome duplication. Genome Biol. 8, R209–R222. (10.1186/gb-2007-8-10-r209) 17916239 PMC2246283

[16] Makino T, McLysaght A 2012 Positionally-biased gene loss after whole genome duplication: evidence from human, yeast and plant. Genome Res. 22, 2427–2435. (10.1101/gr.131953.111) 22835904 PMC3514672

[17] Dolinski K, Botstein D 2007 Orthology and functional conservation in eukaryotes. Annu. Rev. Genet. 41, 465–507. (10.1146/annurev.genet.40.110405.090439) 17678444

[18] Tehlivets O, Scheuringer K, Kohlwein SD 2007 Fatty acid synthesis and elongation in yeast. Biochim. Biophys. Acta 1771, 255–270. (10.1016/j.bbalip.2006.07.004) 16950653

[19] Ding J, Huang X, Zhang L, Zhao N, Yang D, Zhang K 2009 Tolerance and stress response to ethanol in the yeast *Saccharomyces cerevisiae*. Appl. Microbiol. Biotechnol. 85, 253–263. (10.1007/s00253-009-2223-1) 19756577

[20] Baudin A, Ozier-Kalogeropoulos O, Denouel A, Lacroute F, Cullin C 1993 A simple and efficient method for direct gene deletion in *Saccharomyces cerevisiae*. Nucleic Acids Res. 21, 3329–3330. (10.1093/nar/21.14.3329) 8341614 PMC309783

[21] Wach A, Brachat A, Pohlmann R, Philippsen P 1994 New heterologous modules for classical or PCR-based gene disruptions in *Saccharomyces cerevisiae*. Yeast 10, 1793–1808. (10.1002/yea.320101310) 7747518

[22] Gietz RD, Schiestl RH, Willems AR, Woods RA 1995 Studies on the transformation of intact yeast cells by the LiAc/SS-DNA/PEG procedure. Yeast 11, 355–360. (10.1002/yea.320110408) 7785336

[23] Teixeira MC et al. 2006 The YEASTRACT database: a tool for the analysis of transcription regulatory associations in *Saccharomyces cerevisiae*. Nucleic Acids Res. 34(Database issue), D446–D451. (10.1093/nar/gkj013) 16381908 PMC1347376

[24] Avelar AT, Perfeito L, Gordo I, Ferreira MG 2013 Genome architecture is a selectable trait that can be maintained by antagonistic pleiotropy. Nat. Commun. 4, 2235. 23974178 10.1038/ncomms3235

[25] Lang GI, Botstein D, Desai MM 2011 Genetic variation and the fate of beneficial mutations in asexual populations. Genetics 188, 647–661. (10.1534/genetics.111.128942) 21546542 PMC3176544

[26] Piatkowska EM, Naseeb S, Knight D, Delneri D 2013 Chimeric protein complexes in hybrid species generate novel phenotypes. PLoS Genet. 9, e1003836 (10.1371/journal.pgen.1003836) 24137105 PMC3789821

[27] Hooks KB, Naseeb S, Parker S, Griffiths-Jones S, Delneri D 2016 Novel intronic RNA structures contribute to maintenance of phenotype in *Saccharomyces cerevisiae*. Genetics 203, 1469–1481. (10.1534/genetics.115.185363) 27194751 PMC4937481

[28] Naseeb S, Delneri D 2012 Impact of chromosomal inversions on the yeast DAL cluster. PLoS ONE 7, e42022 (10.1371/journal.pone.0042022) 22916115 PMC3419248

[29] Fujita S, Hashimoto T 2000 DNA fingerprinting patterns of *Candida* species using HinfI endonuclease. Int. J. Syst. Evol. Microbiol. 50, 1381–1389. (10.1099/00207713-50-3-1381) 10843084

[30] Langmead B, Salzberg SL 2012 Fast gapped-read alignment with Bowtie 2. Nat. Methods 9, 357–359. (10.1038/nmeth.1923) 22388286 PMC3322381

[31] Li H et al. 2009 The sequence alignment/map format and SAMtools. Bioinformatics 25, 2078–2079. (10.1093/bioinformatics/btp352) 19505943 PMC2723002

[32] McKenna A et al. 2010 The Genome Analysis Toolkit: a MapReduce framework for analyzing next-generation DNA sequencing data. Genome Res. 20, 1297–1303. (10.1101/gr.107524.110) 20644199 PMC2928508

[33] Ashburner M et al. 2000 Gene ontology: tool for the unification of biology. The Gene Ontology Consortium. Nat. Genet. 25, 25–29. (10.1038/75556) 10802651 PMC3037419

[34] Naseeb S, Carter Z, Minnis D, Donaldson I, Zeef L, Delneri D 2016 Widespread impact of chromosomal inversions on gene expression uncovers robustness via phenotypic buffering. Mol. Biol. Evol. 33, 1679–1696. (10.1093/molbev/msw045) 26929245 PMC4915352

[35] Anders S, Pyl PT, Huber W 2014 HTSeq: a Python framework to work with high-throughput sequencing data. Bioinformatics 2014, btu638. 10.1093/bioinformatics/btu638 PMC4287950 25260700

[36] Xie C, Tammi MT 2009 CNV-seq, a new method to detect copy number variation using high-throughput sequencing. BMC Bioinformatics 10, 80 (10.1186/1471-2105-10-80) 19267900 PMC2667514

[37] Delneri D, Tomlin GC, Wixon JL, Hutter A, Sefton M, Louis EJ, Oliver SG 2000 Exploring redundancy in the yeast genome: an improved strategy for use of the cre-loxP system. Gene 252, 127–135. (10.1016/S0378-1119(00)00217-1) 10903444

[38] Carter Z, Delneri D 2010 New generation of loxP-mutated deletion cassettes for the genetic manipulation of yeast natural isolates. Yeast 27, 765–775. (10.1002/yea.1774) 20641014

[39] Delneri D, Colson I, Grammenoudi S, Roberts IN, Louis EJ, Oliver SG 2003 Engineering evolution to study speciation in yeasts. Nature 422, 68–72. (10.1038/nature01418) 12621434

[40] Xu Z et al. 2009 Bidirectional promoters generate pervasive transcription in yeast. Nature 457, 1033–1037. (10.1038/nature07728) 19169243 PMC2766638

[41] Allshire RC, Karpen GH 2008 Epigenetic regulation of centromeric chromatin: old dogs, new tricks? Nat. Rev. Genet. 9, 923–937. (10.1038/nrg2466) 19002142 PMC2586333

[42] Ohkuni K, Kitagawa K 2012 Role of transcription at centromeres in budding yeast. Transcription 3, 193–197. (10.4161/trns.20884) 22885815 PMC3654769

[43] Ozcan S, Johnston M 1999 Function and regulation of yeast hexose transporters. Microbiol. Mol. Biol. Rev. 63, 554. 10477308 10.1128/mmbr.63.3.554-569.1999 PMC103746

[44] Gasch AP, Spellman PT, Kao CM, Carmel-Harel O, Eisen MB, Storz G, Botstein D, Brown PO 2000 Genomic expression programs in the response of yeast cells to environmental changes. Mol. Biol. Cell 11, 4241–4257. (10.1091/mbc.11.12.4241) 11102521 PMC15070

[45] Causton HC et al. 2001 Remodeling of yeast genome expression in response to environmental changes. Mol. Biol. Cell 12, 323–337. (10.1091/mbc.12.2.323) 11179418 PMC30946

[46] Barrick JE, Yu DS, Yoon SH, Jeong H, Oh TK, Schneider D, Lenski RE, Kim JF 2009 Genome evolution and adaptation in a long-term experiment with Escherichia coli. Nature 461, 1243–1247. (10.1038/nature08480) 19838166

[47] Springer M, Weissman JS, Kirschner MW 2010 A general lack of compensation for gene dosage in yeast. Mol. Syst. Biol. 6, 368 (10.1038/msb.2010.19) 20461075 PMC2890323

[48] Qian W, Liao BY, Chang AY, Zhang J 2010 Maintenance of duplicate genes and their functional redundancy by reduced expression. Trends Genet. 26, 425–430. (10.1016/j.tig.2010.07.002) 20708291 PMC2942974

[49] Gout J-F, Lynch M 2015 Maintenance and loss of duplicated genes by dosage subfunctionalization. Mol. Biol. Evol. 2015, msv095 (10.1093/molbev/msv095) PMC4833079 25908670

[50] Velculescu VE, Zhang L, Zhou W, Vogelstein J, Basrai MA, Bassett DE, Hieter P, Vogelstein B, Kinzler KW 1997 Characterization of the yeast transcriptome. Cell 88, 243–251. (10.1016/S0092-8674(00)81845-0) 9008165

[51] Gottschling D, Aparicio O, Billington B, Zakian V 1990 Position effect at S. cerevisiae telomeres: reversible repression of POL II transcription. Cell 63, 751–762. (10.1016/0092-8674(90)90141-Z) 2225075

[52] Chen S, Krinsky BH, Long M 2013 New genes as drivers of phenotypic evolution. Nat. Rev. Genet. 14, 645–660. (10.1038/nrg3521) 23949544 PMC4236023

[53] Chen S, Ni X, Krinsky BH, Zhang YE, Vibranovski MD, White KP, Long M 2012 Reshaping of global gene expression networks and sex-biased gene expression by integration of a young gene. EMBO J. 31, 2798–2809. (10.1038/emboj.2012.108) 22543869 PMC3380208

[54] Chen S, Zhang YE, Long M 2010 New genes in *Drosophila* quickly become essential. Science 330, 1682–1685. (10.1126/science.1196380) 21164016 PMC7211344

[55] Ross BD, Rosin L, Thomae AW, Hiatt MA, Vermaak D, de la Cruz AF, Imhof A, Mellone BG, Malik HS 2013 Stepwise evolution of essential centromere function in a *Drosophila* neogene. Science 340, 1211–1214. (10.1126/science.1234393) 23744945 PMC4119826

[56] Emerson JJ, Cardoso-Moreira M, Borevitz JO, Long M 2008 Natural selection shapes genome-wide patterns of copy-number polymorphism in *Drosophila melanogaster*. Science 320, 1629–1631. (10.1126/science.1158078) 18535209

[57] Lang GI, Rice DP, Hickman MJ, Sodergren E, Weinstock GM, Botstein D, Desai MM 2013 Pervasive genetic hitchhiking and clonal interference in forty evolving yeast populations. Nature 500, 571–574. (10.1038/nature12344) 23873039 PMC3758440

[58] Wagner A 2000 Inferring lifestyle from gene expression patterns. Mol. Biol. Evol. 17, 1985–1987. (10.1093/oxfordjournals.molbev.a026299) 11110914

[59] Hu XH, Wang MH, Tan T, Li JR, Yang H, Leach L, Zhang RM, Luo ZW 2007 Genetic dissection of ethanol tolerance in the budding yeast *Saccharomyces cerevisiae*. Genetics 175, 1479–1487. (10.1534/genetics.106.065292) 17194785 PMC1840089

[60] Teng X, Hardwick JM 2014 Genome evolution in yeast reveals connections between rare mutations in human cancers. Microb. Cell 1, 206–209. (10.15698/mic2014.06.153) 28357245 PMC5354563

[61] Li WH 1980 Rate of gene silencing at duplicate loci: a theoretical study and interpretation of data from tetraploid fishes. Genetics 95, 237–258. 7429144 10.1093/genetics/95.1.237 PMC1214219

[62] Krebs RA, Feder ME 1997 Deleterious consequences of Hsp70 overexpression in *Drosophila melanogaster* larvae. Cell Stress Chaperones 2, 60 (10.1379/1466-1268(1997)002%3C0060:DCOHOI%3E2.3.CO;2) 9250396 PMC312981

[63] Liu H, Krizek J, Bretscher A 1992 Construction of a GAL1-regulated yeast cDNA expression library and its application to the identification of genes whose overexpression causes lethality in yeast. Genetics 132, 665–673. 1468625 10.1093/genetics/132.3.665 PMC1205205

[64] Guan Y, Dunham MJ, Troyanskaya OG 2007 Functional analysis of gene duplications in *Saccharomyces cerevisiae*. Genetics 175, 933–943. (10.1534/genetics.106.064329) 17151249 PMC1800624

[65] Scannell DR, Byrne KP, Gordon JL, Wong S, Wolfe KH 2006 Multiple rounds of speciation associated with reciprocal gene loss in polyploid yeasts. Nature 440, 341–345. (10.1038/nature04562) 16541074

