# Supplementary material for: Rapid functional and evolutionary changes follow gene duplication in yeast
Source: Proc Biol Sci. 2017 Aug 23;284(1861):20171393. doi: 10.1098/rspb.2017.1393 (PMC5577496; doi:10.1098/rspb.2017.1393)
Supplement: Supplementary file 1 [file rspb20171393supp1.docx]

**Supplementary File 1**

**a. Whole-genome sequencing analysis**

Reads were judged to be of good quality with per base sequence quality >30 across the full length of all reads. No filtering or trimming was applied to reads. Reads from each sample were aligned to the yeast reference genome (UCSC sacCer3) using Bowtie2 (1), with the 'local' alignment method and the 'sensitive' alignment preset (*i.e.* –D 15 –R 2 –N 0 –L 20 –i S,1,0.75). For all samples >93% of paired reads were aligned to the reference genome. Samtools (2) was used for sorting and pileup of the alignment files with default parameters.

Single nucleotide polymorphisms (SNPs) were identified using the Genome Analysis ToolKit (GATK) (3) according to the GATK best practice reccommendations. Alignment files were processed and SNPs identified using standard hard filtering parameters (QD < 2, FS > 60 & MQ < 40) (4, 5). Any SNPs identified in both the ancestral and evolved strains were removed from the analysis. SNPs were annotated using SnpEff and the *S. cerevisiae* annotation database R64-1-1.82 (6). SNPs predicted to cause missense mutations were selected.

Genes containing SNPs in each strain were tested for enrichment of Gene Ontology (GO) terms (7) with annotations and GO terms from the *Saccharomyces* Genome Database. Enrichment was determined using Fisher’s exact test with the FDR correction method of Benjamini and Hochberg (8). Terms were classified as enriched if the corrected *p* < 0.05.

**c. RNAseq and copy number variant analysis analyis**

RNA-Seq reads were aligned to the yeast reference genome using Bowtie2 with the 'local' alignment method and the 'sensitive' alignment preset (*i.e.* –D 15 –R 2 –N 0 –L 20 –i S,1,0.75) (1). SAM format files were converted to BAM format using samtools with default parameters (2).

HT-Seq was used to count reads mapped to genes with a quality score of >20 and according to HT-Seq’s ‘union’ model (9). Differentially expressed (DE) genes were identified using edgeR (10). In this analysis genes with < 10 counts per million in 2 or more samples were excluded. DE was calculated using edgeR's exact test and *p-values* were corrected for FDR (8). Significantly DE genes were those with a corrected *p* < 0.01. Hierarchical clustering of genes based on *log(fold change)* of expression was carried out and groups of genes were tested for enrichment of GO terms (7)

Two of the five biological replicates for each duplication strain evolved in each environment were sequenced, along with two technical replicates for each biological replicate. DE analysis was performed on each biological replicate separately, as each replicate may have diverged, with the technical replicates used to normalise the data. Significant overlap of DE genes between biological replicates was identified using Rank-Rank Hypergeometric Overlap (RRHO) where genes were ranked by *log(fold change)* and a cut off of *p* < 0.05 was used to defined a significant overlap (11). Hierarchical clustering of genes based on *log(fold change)* of expression was used to identify groups of genes showing similar changes in expression across samples. These groups of genes were tested for enrichment of GO terms (7). Enrichment was determined using Fisher’s exact test with FDR (8). Terms were classified as enriched if the corrected *p* < 0.05.

To test the robustness of our results we also analysed biological replicates together. Both methods of data analysis lead to very similar results: for example for the tandem duplicate YPD evolved strains we identified 3,020 and 3,354 DE genes treating biological replicates separately and together, respectively. Out of these DE genes 2,953 (97.7%) are significant (*i.e.* overlapping) in both methods. Moreover we find a strong correlation (R^2^ always > 0.87) in estimated *log(fold change)* for both our analysis methods. Taken together these results indicate that our analysis is robust.

To identify any changes in copy number of *IFA38* after experimental evolution we used CNV-Seq (12) to compare the read-depth between ancestral and evolved strains. We used a window size of 200 bases and *log(fold change)* >= 0.6 to identify significant changes in copy number. Identified changes were subsequently experimentally validated.

**References**

1. Langmead B, Salzberg SL. Fast gapped-read alignment with Bowtie 2. Nature methods. 2012;9(4):357--9.

2. Li H, Handsaker B, Wysoker A, Fennell T, Ruan J, Homer N, et al. The sequence alignment/map format and SAMtools. Bioinformatics. 2009;25(16):2078--9.

3. McKenna A, Hanna M, Banks E, Sivachenko A, Cibulskis K, Kernytsky A, et al. The Genome Analysis Toolkit: a MapReduce framework for analyzing next-generation DNA sequencing data. Genome research. 2010;20(9):1297--303.

4. Auwera GA, Carneiro MO, Hartl C, Poplin R, del Angel G, Levy-Moonshine A, et al. From FastQ data to high-confidence variant calls: the genome analysis toolkit best practices pipeline. Current protocols in bioinformatics. 2013:11--0.

5. DePristo MA, Banks E, Poplin R, Garimella KV, Maguire JR, Hartl C, et al. A framework for variation discovery and genotyping using next-generation DNA sequencing data. Nature genetics. 2011;43(5):491--8.

6. Cingolani P, Platts A, Coon M, Nguyen T, Wang L, Land SJ, et al. A program for annotating and predicting the effects of single nucleotide polymorphisms, SnpEff: SNPs in the genome of Drosophila melanogaster strain w1118; iso-2; iso-3. Fly. 2012;6(2):80-92.

7. Ashburner M, Ball C, Blake J, Botstein D, Butler H, Cherry J, et al. Gene ontology: tool for the unification of biology. The Gene Ontology Consortium. Nature Genetics. 2000;25(1):25--9.

8. Benjamini Y, Hochberg Y. Controlling the false discovery rate: a practical and powerful approach to multiple testing. Journal of the Royal Statistical Society Series B (Methodological). 1995;57(1):289--300.

9. Anders S, Pyl PT, Huber W. HTSeq–A Python framework to work with high-throughput sequencing data. Bioinformatics. 2014:btu638.

10. Robinson MD, McCarthy DJ, Smyth GK. edgeR: a Bioconductor package for differential expression analysis of digital gene expression data. Bioinformatics. 2010;26(1):139--40.

11. Plaisier SB, Taschereau R, Wong JA, Graeber TG. Rank--rank hypergeometric overlap: identification of statistically significant overlap between gene-expression signatures. Nucleic acids research. 2010;38(17):e169--e.

12. Xie C, Tammi MT. CNV-seq, a new method to detect copy number variation using high-throughput sequencing. BMC bioinformatics. 2009;10(1):80.
