# Supplementary material for: Rapid functional and evolutionary changes follow gene duplication in yeast
Source: Proc Biol Sci. 2017 Aug 23;284(1861):20171393. doi: 10.1098/rspb.2017.1393 (PMC5577496; doi:10.1098/rspb.2017.1393)
Supplement: Supplementary Figures and tables Legends [file rspb20171393supp2.docx]

**Supplementary Figure Legends**

Figure S1: Strategy used to engineer strains possessing tandem and non-tandem duplication. (A) Schematic representation of chromosome II showing the location of *IFA38* gene (red colour) and UTR regions (pink) of the wild type strain (co-ordinates: 558632..559848). (B) *IFA38* gene (dark blue) along with its UTRs (light blue) was tagged by a *loxP-KanMX-loxP* resistance marker (green colour) to make a duplication cassette. Artificial copy of *IFA38* gene tagged by a KanMX resistance marker (duplication cassette) was inserted in the genome at tandem position and (C) confirmed by analytical PCR using primer pairs a+b and c+d (D) Artificial copy of *IFA38* gene tagged by a KanMX resistance marker (duplication cassette) was inserted in the genome at non-tandem location and (E) verified by analytical PCR using primer pairs a+f and c+g (F). Small black arrows show primer positions. The colonies tested and the DNA Hyperladder 1000 bp (L) are separated on 1.5% agarose gel (D and F).

**Figure S2: Fitness of *IFA38* deletion mutant in YPD and YPD+ethanol medium.** The fitness of *IFA38* homozygous deletion mutant (blue box) was found to be lower compared to the wild type BY4743 strain (red box) in ethanol containing medium. The box plots represents the means of the area under the growth curve (AUC) as determined by the R grofit package. The small white circles show the distribution of five technical replicates. Significance is estimated by paired t-test (p < 0.01).

**Figure S3: Expression levels of *IFA38* in the BY4743 (wild type), IFA38-t and IFA38-nt strains.** Real time PCR shows the expression of *IFA38* in YPD **(A)**, YPD + 7% ethanol **(B)** and YP + 2% glycerol **(C)**. Relative normalized fold expression was calculated by using ΔΔCt method and *ACT1* as a reference gene. Red, Blue and green boxes represents the BY4743 control, tandem (IFA38-t) and non-tandem (IFA38-nt) duplicated strains, respectively. Error bars are from three technical replicas for each of the five independent biological samples.

Figure S4: Competitive fitness in YPD medium of evolved strains: Fitness coefficients of evolved BY4743 (A), IFA38-t (B) and IFA38-nt (C) duplicates relative to their respective ancestral strains competed in YPD medium. The results are from strains that were evolved in YPD (broken line), YPD+ethanol (dotted line) and glycerol (solid line) medium. T1, T2 and T3 represent three different points of cell count after every 10 generations. The error bars represent the average of three technical replicas of five independent biological replicas. Error bars are at 95% confidence intervals. “t” and “nt” refers to tandem and non-tandem duplicates, respectively.

**Figure S5: Competitive fitness in YPD + 7% ethanol medium of evolved strains:** Fitness coefficients of evolved BY4743 **(A)**, IFA38-t **(B)** and IFA38-nt **(C)** duplicates relative to their respective ancestral strains competed in YPD + 7% ethanol medium. The results are from strains that were evolved in YPD (broken line), YPD+ethanol (dotted line) and glycerol (solid line) medium. T1, T2 and T3 represent three different points of cell count after every 10 generations. The error bars represent the average of three technical replicas of five independent biological replicas. Error bars are at 95% confidence intervals. “t” and “nt” refers to tandem and non-tandem duplicates respectively.

Figure S6: Competitive fitness in YP + 2% Glycerol medium of evolved strains. Fitness coefficients of evolved BY4743 (A), IFA38-t (B) and IFA38-nt (C) duplicates relative to their respective ancestral strains competed in YP+glycerol medium. The results are from strains that were evolved in YPD (broken line), YPD+ethanol (dotted line) and glycerol (solid line) medium. T1, T2 and T3 represent three different points of cell count after every 10 generations. The error bars represent the average of three technical replicas of five independent biological replicas. Error bars are at 95% confidence intervals.

**Figure S7: Number of common and unique genes that accumulate SNPs for each strain evolved in EtOH, YPD and glycerol.** Venn diagrams show the number of unique and overlapping genes that accumulate SNPs across 3 environments for **(A)** the wild-type strain, **(B)** the tandem duplication and **(C)** the non-tandem duplication.

**Figure S8: Evidence of duplicate loss in glycerol evolved strains from next-generation sequencing data.**  Non-tandem biological replicate 1 show a significant reduction of read depth after experimental evolution in the region of *IFA38* relative to the ancestral strain. The blue broken lines represent the genomic position of *IFA38*. Each point represents a genomic window and the colouration shows the significance of any change in read depth.

Figure S9: Analytical PCR to confirm the presence of original *IFA38* gene in ancestral and glycerol evolved non-tandem duplicates. (A) A diagrammatic view of the original gene (red) and engineered copy (blue) of *IFA38* on the chromosome and the specific primers (small black arrows) used for PCR. (B) 1.5% (w/v) agarose gel representing the colonies of ancestral and glycerol evolved duplicated strains confirmed by primer pair a+b giving a product of expected band size 318bp. (C) 1.5% (w/v) agarose gel representing the colonies of ancestral and glycerol evolved duplicated strains confirmed by primer pair c+d giving a product of expected band size 838bp.

**Supplementary Tables**

**Table S1**: The set primers used to amplify the duplication cassette.

**Table S2**: The set of checking primers used in analytical PCR

**Table S3:** Codon W analysis of *IFA38* and duplicate *IFA38* sequence with substitutions.

**Table S4:** The set of primers used for Real Time PCR.

**Table S5:** The enriched GO terms for consistently up- and down-regulated set of genes between evolved duplicate and wild-type strains.

**Table S6:** Full lists of DE genes immediately after experimental duplication and after long-term evolution.

**Table S7:** Identified single nucleotide polymorphisms (SNPs) after experimental evolution.

**Table S8**: Enriched GO terms for genes with identified SNPs after experimental evolution.
