# Supplementary figures and images for: Rapid functional and evolutionary changes follow gene duplication in yeast
Source: Proc Biol Sci. 2017 Aug 23;284(1861):20171393. doi: 10.1098/rspb.2017.1393 (PMC5577496; doi:10.1098/rspb.2017.1393)

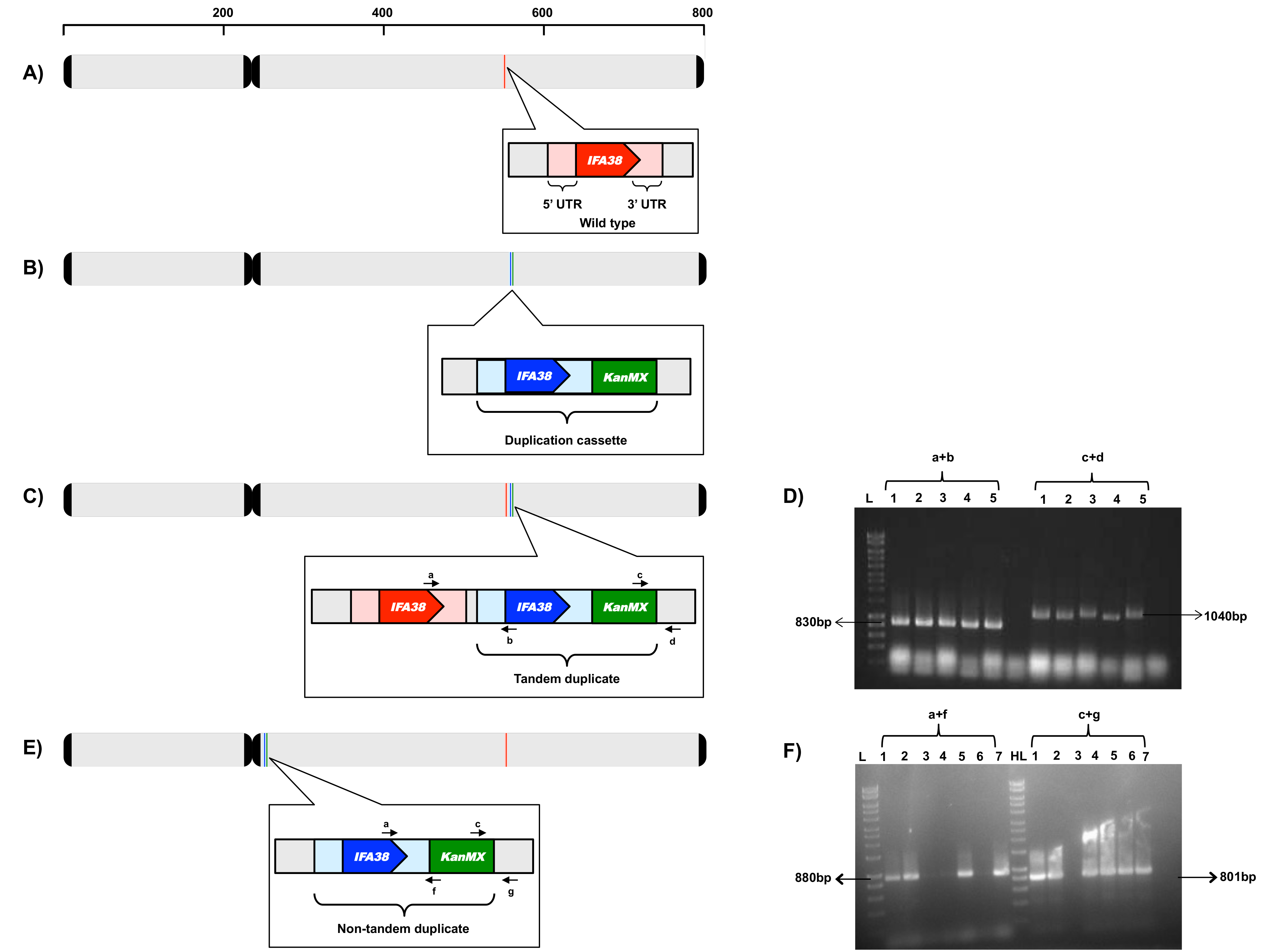

Supplement: Figure S1 [file rspb20171393supp3.tif]

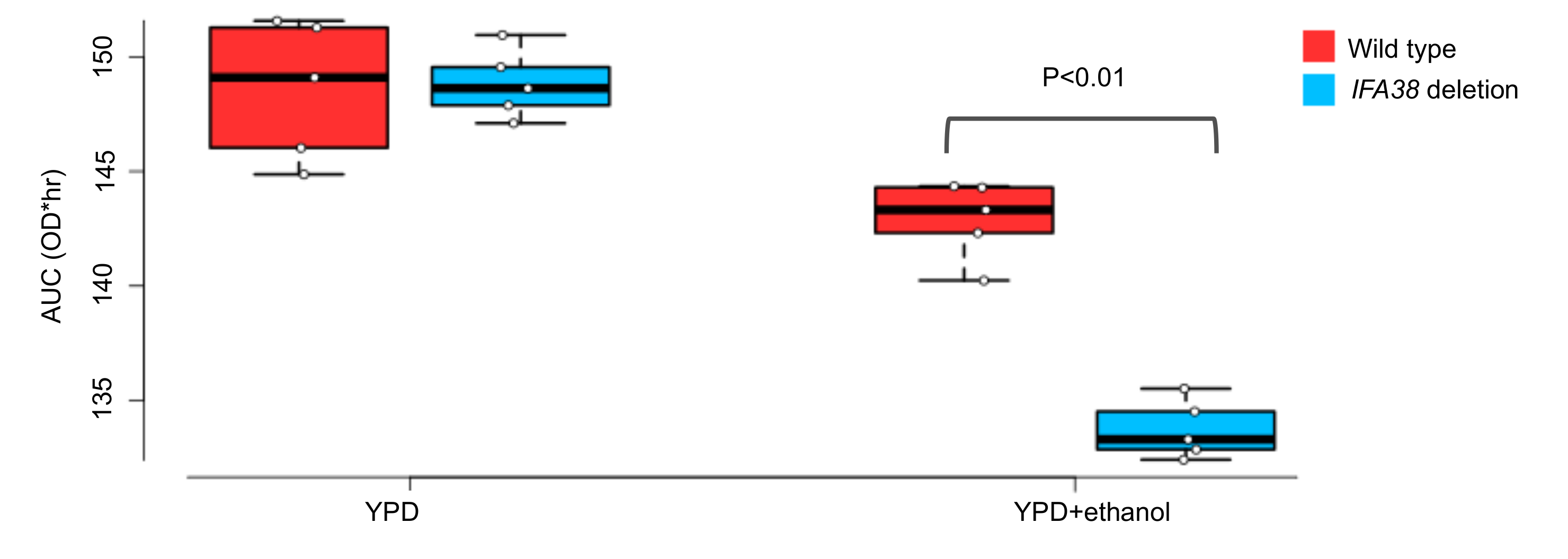

Supplement: Figure S2 [file rspb20171393supp4.tif]

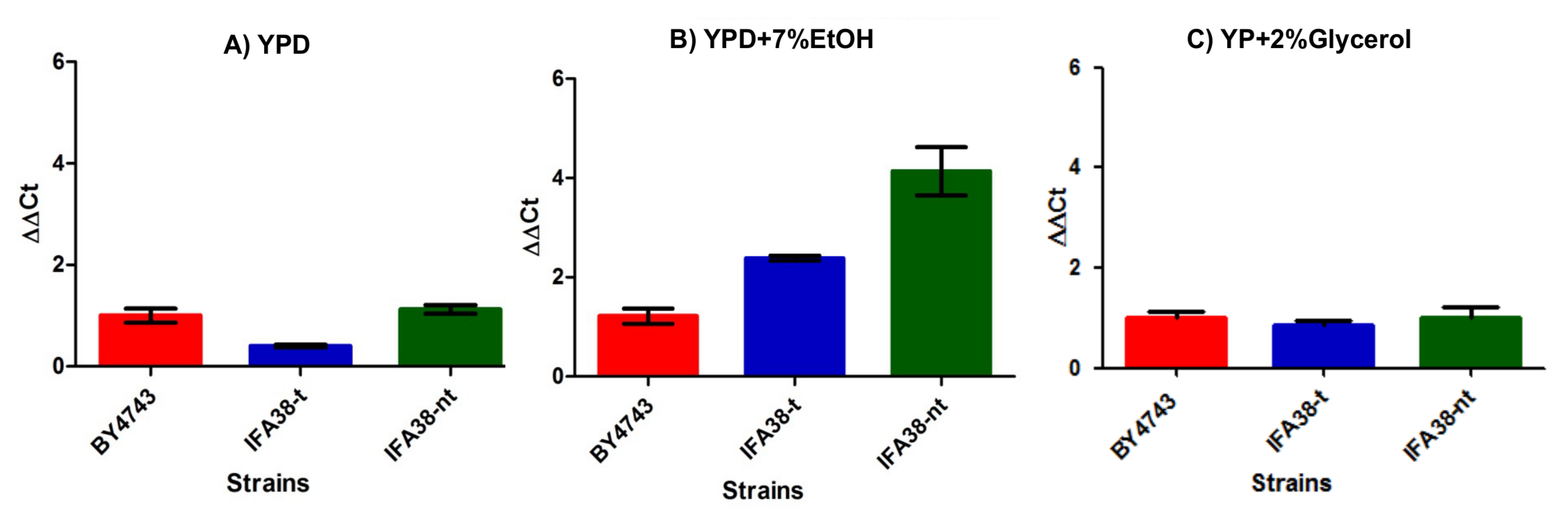

Supplement: Figure S3 [file rspb20171393supp5.tif]

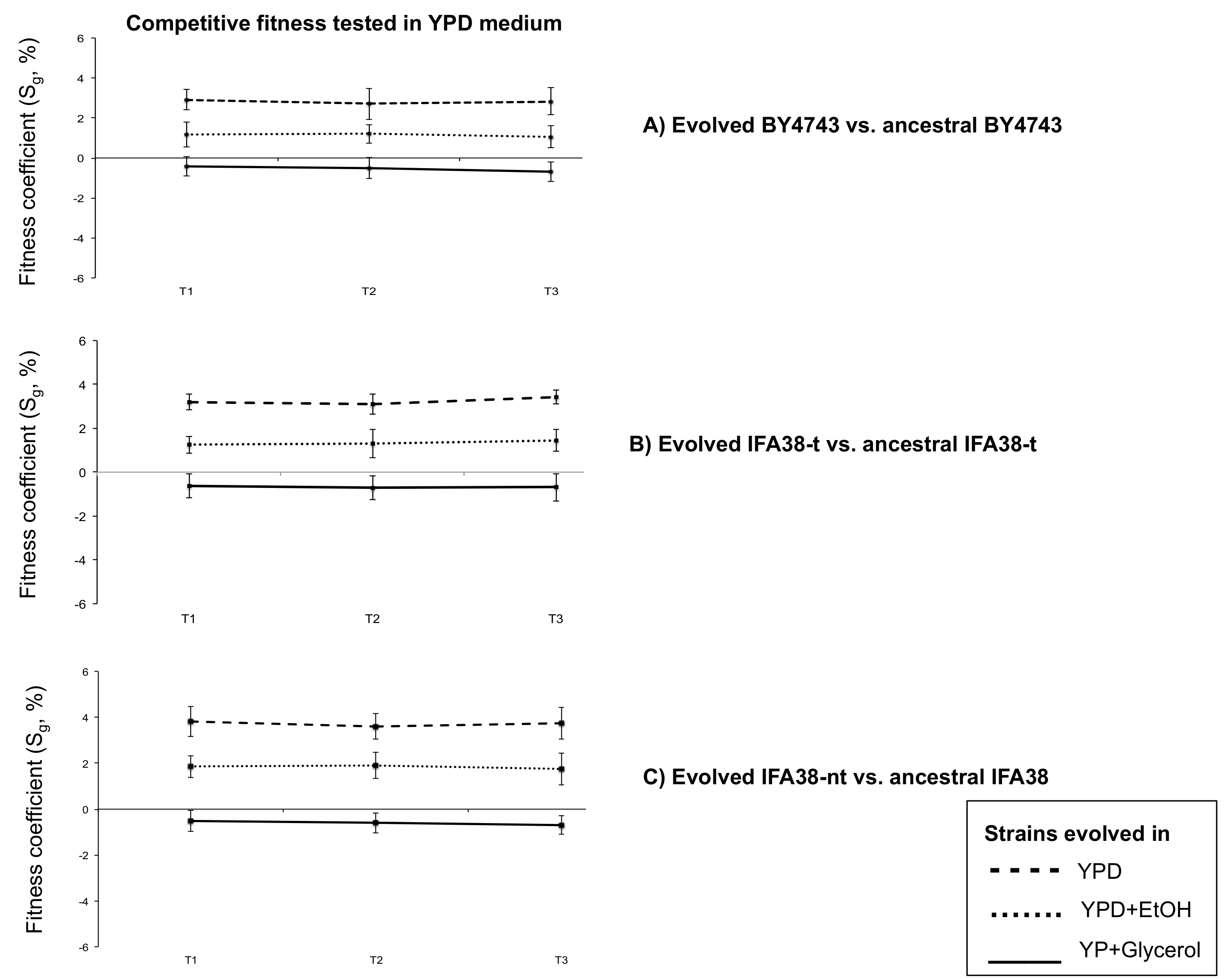

Supplement: Figure S4 [file rspb20171393supp6.tif]

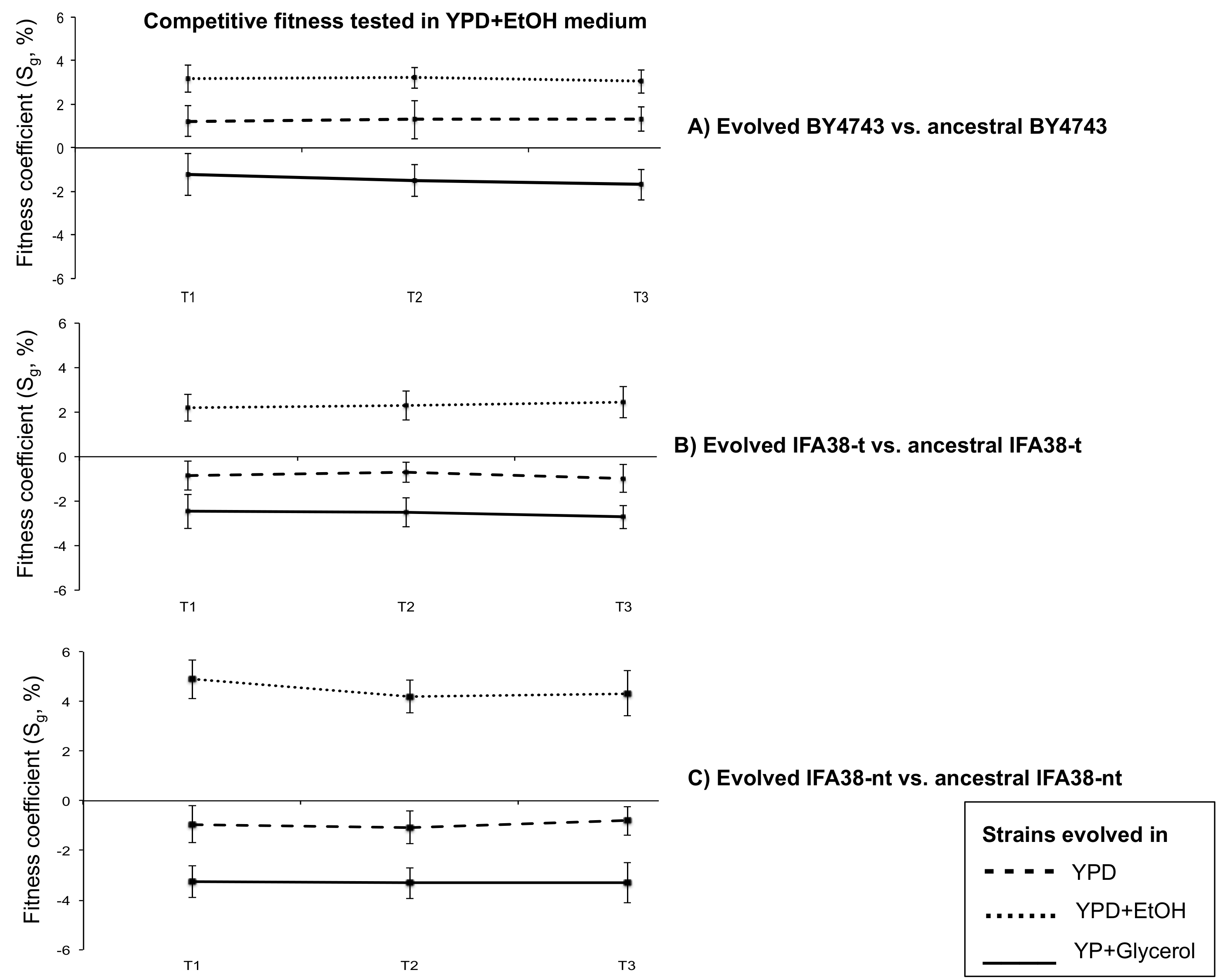

Supplement: Figure S5 [file rspb20171393supp7.tif]

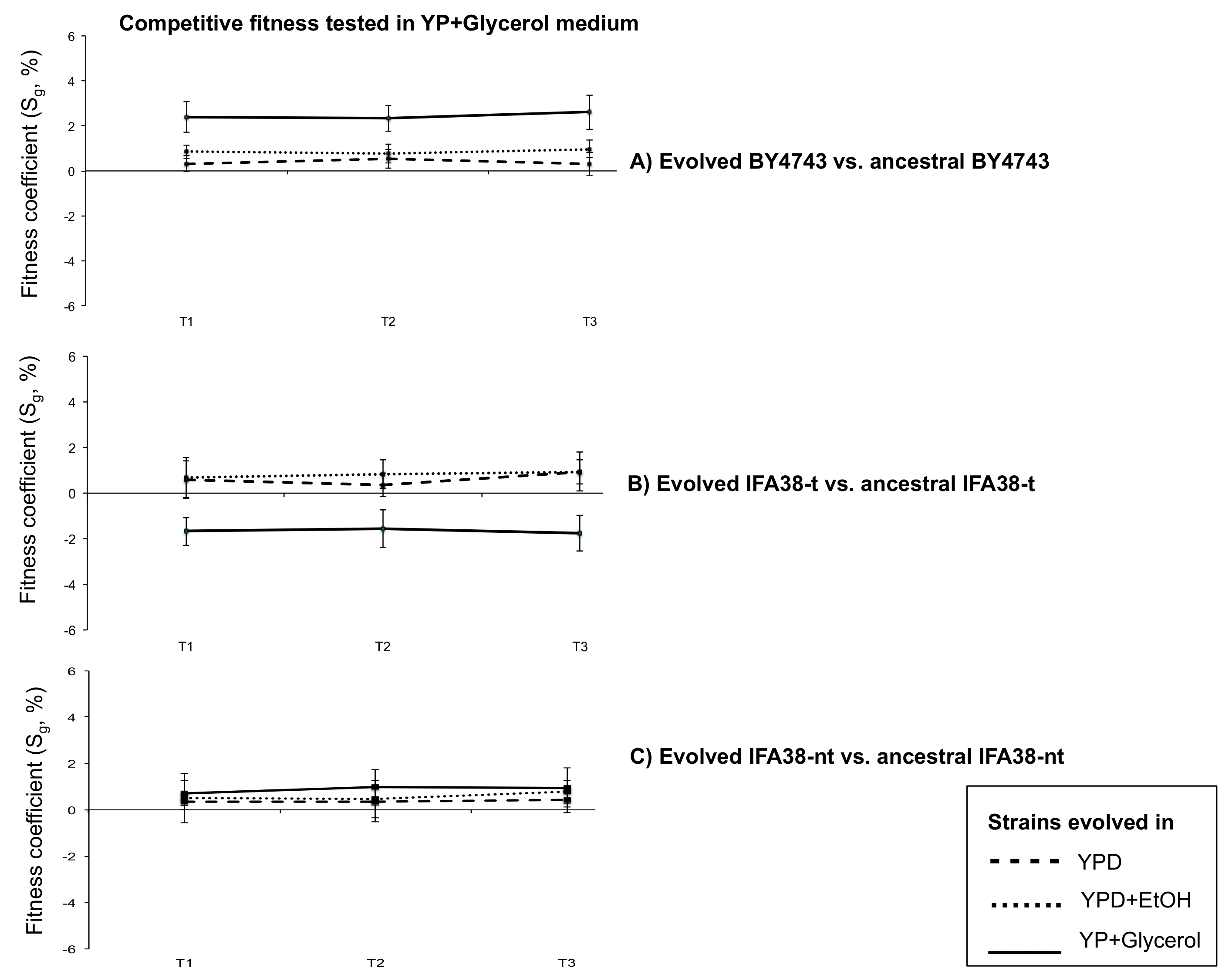

Supplement: Figure S6 [file rspb20171393supp8.tif]

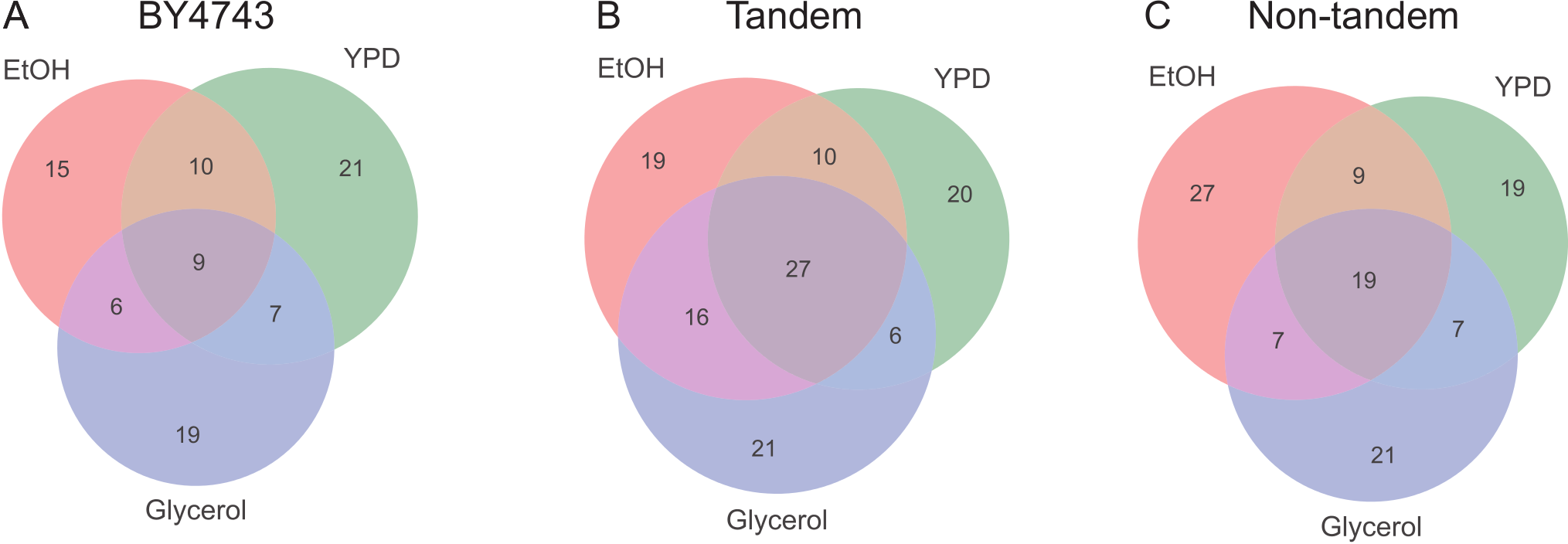

Supplement: Figure S7 [file rspb20171393supp9.tif]

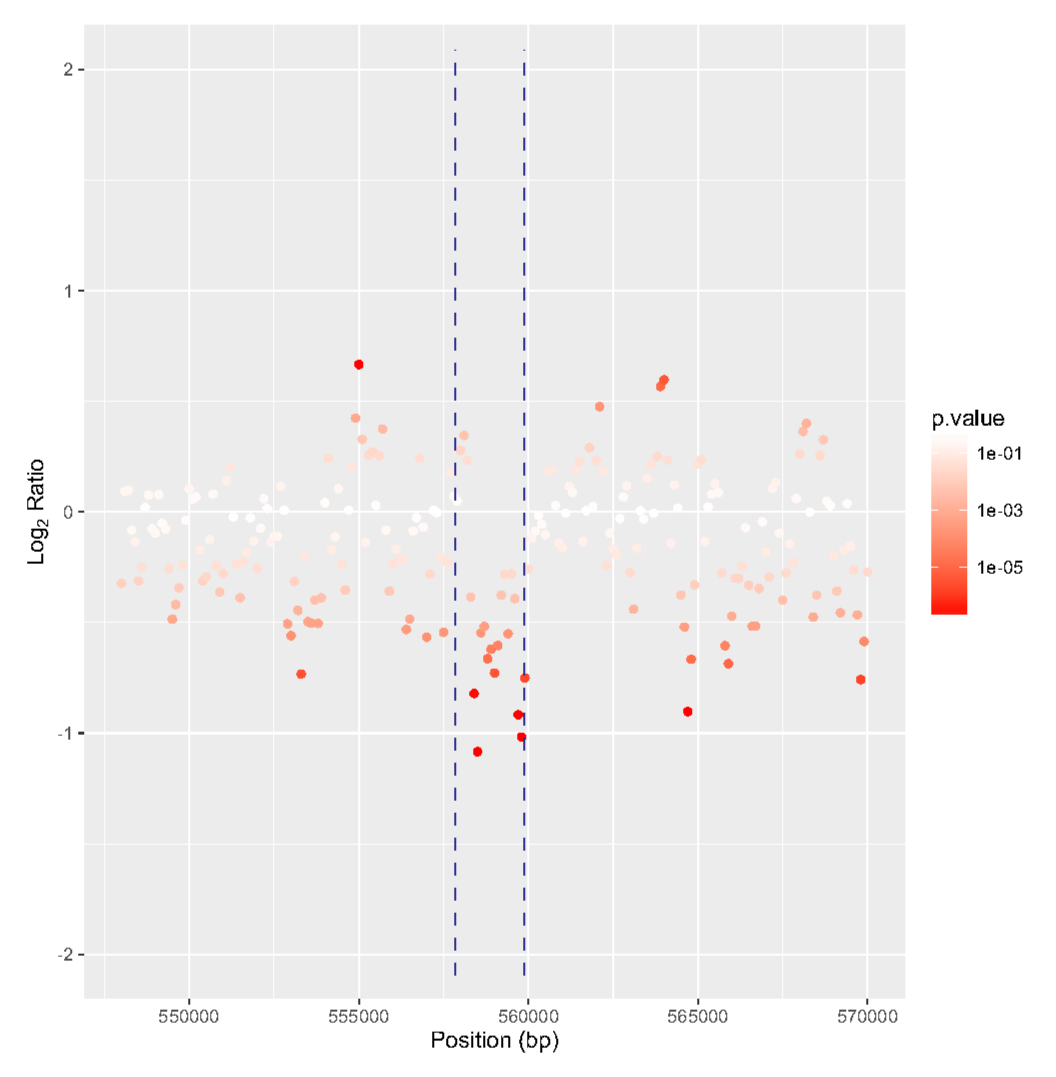

Supplement: Figure S8 [file rspb20171393supp10.tif]

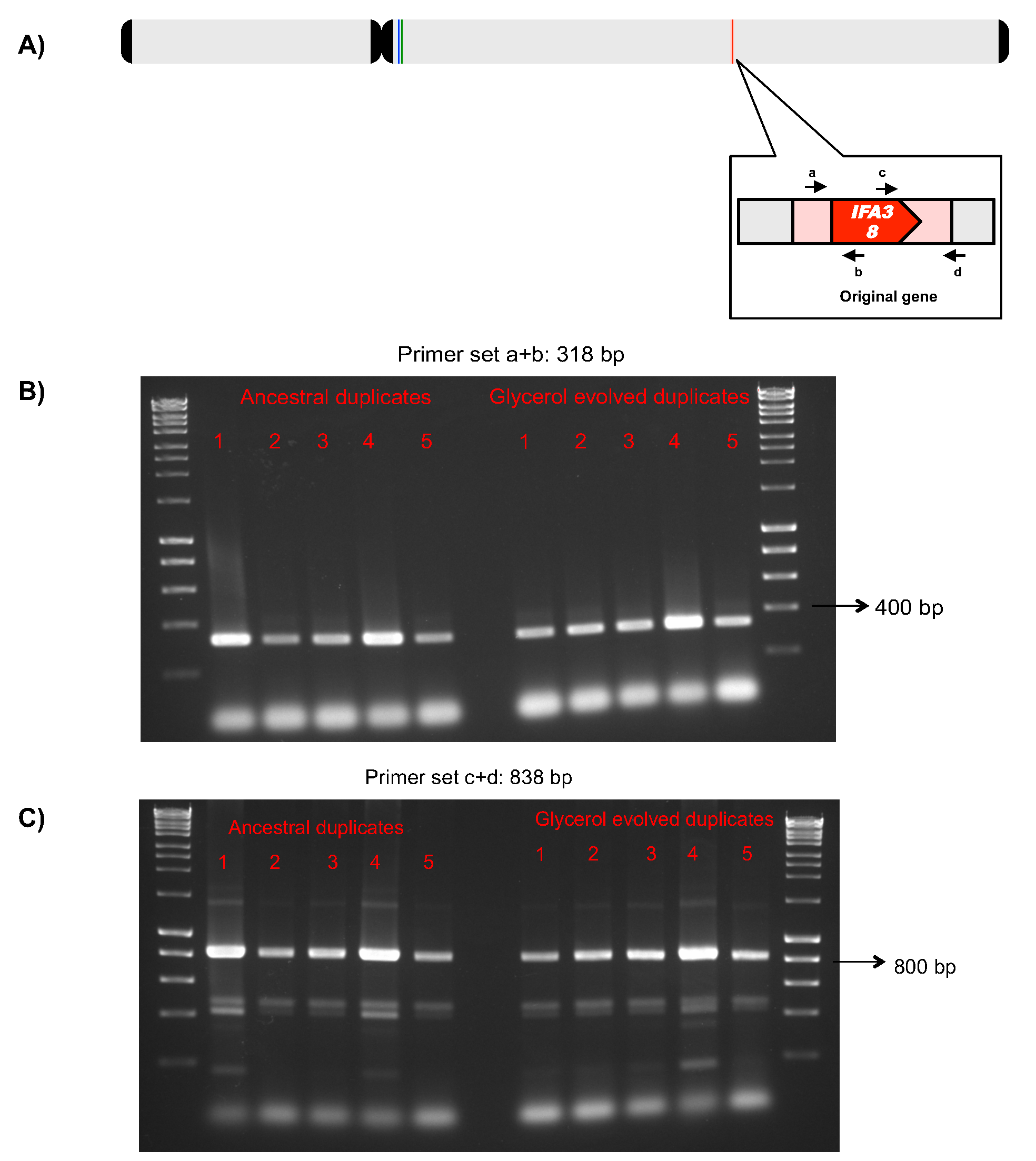

Supplement: Figure S9 [file rspb20171393supp11.tif]
