# Supplementary material for: Rapid functional and evolutionary changes follow gene duplication in yeast
Source: Proc Biol Sci. 2017 Aug 23;284(1861):20171393. doi: 10.1098/rspb.2017.1393 (PMC5577496; doi:10.1098/rspb.2017.1393)
Supplement: Table S1 [file rspb20171393supp12.docx]

**Table S1. The set primers used to amplify the duplication cassette**

| **Primer Name** | **Sequence 5’-3’** | **Tm(^o^C)** |
| --- | --- | --- |
| IFA38-kanMX.amp-F | AGGGCAACAAACGCCAAAAATAAAAACGAACCTAAATGAGAAAAAcgccagctgaagcttcgta | 69.5 |
| IFA38-kanMX.amp-R | CAATTTTTTTTTTTGAGTTCTTGTTGTTTTCCAAATTTTTCTCTTCACTATAGGGAGACCGGCAG | 66.2 |
| IFA38-dup(t).amp-F | GTGGAAGGACCAAGTCCTCTTGAAAGAAAATTTAATACTGCTTTTTATTTTTCGCTTTTTTAGAG | 65.3 |
| IFA38-dup(t).amp-R | AGCTGATGTGCTGGCTTGAATATTTTTTCCAATATGATATTCTGGCACTATAGGGAGACCGGCAG | 68.9 |
| IFA38-dup(nt).amp-F | GAAGTTTTTCACAAGCGGAATAAGTGATACCATTCATAAAATATCATATCTATCTATCTATATTTTTCGCTTTTTTAGAG | 64.8 |
| IFA38-dup(nt).amp-R | ACAGAAAGGCTTAAAAGGAAAGGCATTTATTTTCGTACCATATATCTCACAAAAATAGACACTATAGGGAGACCGGCAG | 68.3 |
