# Supplementary material for: Rapid functional and evolutionary changes follow gene duplication in yeast
Source: Proc Biol Sci. 2017 Aug 23;284(1861):20171393. doi: 10.1098/rspb.2017.1393 (PMC5577496; doi:10.1098/rspb.2017.1393)
Supplement: Table S2 [file rspb20171393supp13.docx]

**Table S2. The set of checking primers used in analytical PCR**

| **Primer Name** | **Sequence 5’-3’** | **Tm(^o^C)** |
| --- | --- | --- |
| IFA38-dup(t).chk-F | ACAGTGGTTCGAAATCATTC | 55 |
| IFA38-dup(t).chk-R | CGACTACTCTTTGACCTTGG | 52 |
| kanMX.chk-F | TCGTCACTCATGGTGATTTC | 52 |
| kanMX.chk-R | AACGTGAGTCTTTTCCTTACC | 52 |
| IFA38-dup(nt).chk-F | TCCAAGGAGGAGACTTTTGG | 55 |
| IFA38-dup(nt).chk-R | CCAATTTAGATTGCGTTCTC | 55 |
