# Supplementary material for: Rapid functional and evolutionary changes follow gene duplication in yeast
Source: Proc Biol Sci. 2017 Aug 23;284(1861):20171393. doi: 10.1098/rspb.2017.1393 (PMC5577496; doi:10.1098/rspb.2017.1393)
Supplement: Table S4 [file rspb20171393supp15.docx]

**Table S3. The set of primers used for Real Time PCR**

| **Primer Name** | **Sequence 5’-3’** | **Tm(^o^C)** |
| --- | --- | --- |
| IFA38-RT(F) | AATTGTGTGCGCAGTTACCA | 56 |
| IFA38-RT(R) | CAAACCACGAGTACCCGACT | 57 |
